# Burden and Trends of Gastroenteritis in Adults in the United States: Insights From CDC WONDER 2018–2023 Surveillance Data

**DOI:** 10.7759/cureus.89225

**Published:** 2025-08-01

**Authors:** Judah K Okechukwu, Oksana Polna, Tochukwu W Okahia, Okelue E Okobi, Stanley Ezulike, Ebere M Nwachukwu, Kingsley O Ozojide

**Affiliations:** 1 Internal Medicine, Ivan-Frankivska National Medical University, Ivano-frankivska, UKR; 2 Internal Medicine, Ivan-Frankivska National Medical University, Ivano-Frankivsk, UKR; 3 Psychiatry, Leeds and York Partnership NHS Foundation Trust, Leeds, GBR; 4 Family Medicine, Larkin Community Hospital Palm Springs Campus, Miami, USA; 5 Family Medicine, IMG Research Academy and Consulting, LLC, Homestead, USA; 6 Internal Medicine, Chukwuemeka Odumegwu Ojukwu University Teaching Hospital, Awka, NGA; 7 Kinesiology - Exercise Science, Georgia Southern University, Statesboro, USA; 8 Public Health, Nottingham Trent University, Nottingham, GBR

**Keywords:** a09, cdc wonder, crude mortality rate, gastroenteritis, infectious diseases, mortality

## Abstract

Background

Gastroenteritis remains a significant cause of morbidity and mortality, particularly among older adults. Despite advances in sanitation and healthcare access, infectious gastroenteritis continues to impact vulnerable populations in the United States (US). This study aimed to assess the burden and temporal trends of gastroenteritis-related mortality among US adults aged 45-84 years over a recent six-year period.

Methods

We conducted a retrospective, descriptive analysis using mortality data from the CDC WONDER (Wide-ranging ONline Data for Epidemiologic Research) Multiple Cause of Death database for the years 2018 to 2023. Deaths attributed to gastroenteritis of infectious origin were identified using the ICD-10 code A09. Variables extracted included year of death, sex, single-year age, Hispanic origin, population estimates, and crude mortality rates. Analysis was restricted to adults aged 45-84 years, and results were stratified by age group, sex, and year. Descriptive statistics and trend summaries were generated using Stata 18.

Results

A clear age-related increase in gastroenteritis mortality was observed. Among individuals aged 75-84 years, females had the highest crude mortality rates, rising from 4.54 per 100,000 in 2018 to 5.31 per 100,000 in 2023. Males in the same age group showed relatively stable rates, ranging from 3.66 to 4.41 per 100,000. Females also exhibited higher mortality than males in the 65-74 year group across all years. Data were limited for the 45-64 age group in several years due to suppression or unreliability. Overall, the burden of gastroenteritis mortality remained concentrated among older adults, with notable increases among elderly women.

Conclusion

Despite overall improvements in public health, gastroenteritis-related mortality remains a persistent concern for aging US populations. The observed sex and age disparities highlight the need for targeted prevention, early intervention, and enhanced infection control strategies, particularly in long-term care settings. Ongoing surveillance is essential to monitor evolving trends and guide resource allocation.

## Introduction

Gastroenteritis, or stomach flu, is a major public health concern noted by inflammation of the large intestine and stomach, which causes symptoms of diarrhea, vomiting, stomach ache, and dehydration [1]. Gastroenteritis is sometimes considered to be a mild, self-limiting disease; however, it may result in severe complications, including death, especially in vulnerable groups, including the elderly, immunocompromised people, and individuals with concomitant diseases [2, 3]. Gastroenteritis burden was experienced differently in the United States over time [4]. This has been largely attributed to changes in demographics, pathogen prevalence, disparity in access to healthcare, and advancements in diagnosis and reporting measures [5].

Concern has been raised in the last 20 years over the re-emergence of some gastroenteritis-causing pathogens, namely *Clostridioides difficile *(*C. difficile*), norovirus, and some *Escherichia coli *(*E. coli*) strains [6, 7]. More specifically, *C. difficile* has risen to the top in terms of gastroenteritis-related deaths, particularly in healthcare facilities, mainly among the geriatric population and in hospitals [8]. At the same time, the enhancements in public health surveillance and laboratory diagnostics have helped in the improved reporting and recording of gastroenteritis cases [9]. However, the reports on the real mortality burden remain unequal because of under-reporting or misreporting of gastroenteritis cases on death certificates [10].

The Centers for Disease Control and Prevention (CDC) Wide-ranging ONline Data for Epidemiologic Research (WONDER) platform offers a valuable tool for evaluating national mortality trends and burden of disease over time [11]. Utilizing death certificate data across the United States, CDC WONDER provides a standardized and comprehensive view of cause-specific mortality across various demographics, geographic regions, and periods [12]. Through this platform, it becomes possible to explore temporal shifts in gastroenteritis-related mortality and identify vulnerable populations most at risk [13].

Other studies have tended to present pediatric subjects or those of particular outbreaks, although little thorough research has been done on the long-term trends of gastroenteritis in adults [14]. Moreover, seasonal distributions, by age group, sex, race, and spatial distributions, can provide insights into inequalities, which provide a basis for focused preventive measures and preventive policymaking [15].

Historically, in the United States, the burden of gastroenteritis has received less public attention than other chronic or infectious diseases [16]. Nevertheless, surveillance data show that the condition has significant health and economic impacts. A total of millions of Americans are affected per year, resulting in hundreds of thousands of emergency department visits and thousands of hospitalizations [17]. The condition is not only burdening the health system but also causes much loss in productivity, through absenteeism and decreased workplace efficiency [18]. Due to the changing risk factors, it is important to monitor gastroenteritis mortality continuously, which is critical to determining the public health approaches and distribution of healthcare resources [19]. This study aims to assess the burden and temporal trends of gastroenteritis-related mortality among adults in the United States from 2018-2023 using CDC WONDER mortality data [20-21]. By analyzing patterns across demographic groups and time periods, this research seeks to provide deeper insights into the epidemiology of gastroenteritis in adult populations.

## Materials and methods

Study design and data source

This was a retrospective, population-based descriptive study utilizing publicly available national mortality data from the CDC WONDER system [21]. Data were extracted from the Multiple Cause of Death database, which compiles death certificate information filed in all 50 states and the District of Columbia. The dataset includes demographic characteristics, underlying causes of death, and population estimates by year, sex, race/ethnicity, and age. For this study, we queried records from 2018 to 2023, covering a six-year surveillance period.

Case definition and variable selection

Gastroenteritis-related deaths were identified using the International Classification of Diseases, Tenth Revision (ICD-10) code A09 (diarrhea and gastroenteritis of presumed infectious origin) as the underlying cause of death. The analysis was limited to adults in the United States aged 45-84 years, grouped into three age categories: 45-64 years, 65-74 years, and 75-84 years. This age restriction was chosen due to the low frequency and unreliable nature of deaths among younger adults, as per CDC WONDER suppression thresholds. Thus, adults below 45 were excluded, as gastroenteritis-linked deaths in the group are rare and often suppressed in CDC WONDER as a result of the small counts, which limits data reliability. Consequently, persons aged above 84 years were excluded due to aggregation issues and potential misclassification in mortality records. The chosen bands are aligned with standard CDC reporting practices, enabling the presentation of stable, interpretable trends across middle-aged, early older, and advanced older adult populations. Furthermore, the extracted variables for this study included the year of death (ranging from 2018 to 2023), sex (categorized as male or female), and age, which was originally provided in single-year increments and subsequently recoded into broader 10- or 20-year age groups for analytical clarity. Additional variables obtained from the CDC WONDER database included Hispanic origin, the number of deaths attributed to gastroenteritis (ICD-10 code A09) as the underlying cause, annual population estimates corresponding to each demographic subgroup, and the crude mortality rate per 100,000 population. These variables enabled the stratification of gastroenteritis mortality trends by age, sex, and year to better understand the burden and demographic distribution of deaths over the six years. Records labeled “Unreliable” or “Not Applicable” for the population or crude rate variables were excluded from quantitative analyses. The data were extracted from the publicly accessible and de-identified CDC WONDER platform, which permits use of ICD codes without special permission, in accordance with its data use agreement.

Data management and statistical analysis

Data were downloaded in tab-delimited format from the CDC WONDER interface and imported into Stata 18.0 (StataCorp LLC, College Station, TX) for cleaning and statistical analysis. Age was recoded from a single-year format into the three predefined age groups. Observations with “unreliable” population counts or “not applicable” rates were filtered prior to analysis to ensure consistency. Descriptive statistics were calculated to summarize total deaths, population counts, and crude mortality rates per 100,000 population, stratified by age group, sex, and year. Results were tabulated and visually represented using bar charts to compare rates across demographic strata. Due to the aggregate and descriptive nature of the study, no inferential statistical tests were conducted. Moreover, despite the reporting of co-occurring conditions in certain cases, they were excluded in the primary analysis unless gastroenteritis was specified as the main cause. It is noteworthy that, even as the analysis is limited to descriptive statistics as a result of the dataset's aggregate nature, no inferential tests were conducted. 

Ethical considerations

This study used de-identified, publicly available mortality data from the CDC WONDER portal. As such, it did not involve human subjects or patient identifiers and was exempt from institutional review board (IRB) approval in accordance with federal regulations and the United States Department of Health and Human Services policy on the use of publicly available data.

## Results

Table 1 below summarizes the total number of gastroenteritis-related deaths, corresponding adult population, and crude mortality rates per 100,000 population across three age groups (45-64, 65-74, and 75-84 years) by sex, based on pooled national mortality data from CDC WONDER for the years 2018 through 2023. The analysis was restricted to adults aged 45-84 years due to data reliability constraints and to focus on the age groups where most gastroenteritis deaths occurred.

**Table 1:** Gastroenteritis mortality by age group and sex among adults in the United States, 2018–2023 Crude mortality rate per 100,000 population, calculated as (deaths/population) × 100,000. Data sourced from CDC WONDER, UCD–ICD-10 code A09 (gastroenteritis of infectious origin).

Male				Female		
Age group	Deaths	Population	Crude rate	Deaths	Population	Crude rate
45-64 years	49	3,525,877	1.39	276	22,895,880	1.21
65-74 years	690	38,074,684	1.81	1640	79,396,342	2.07
75-84 years	1348	33,873,870	3.98	2455	49,933,375	4.92

Across all age categories, both the absolute number of deaths and the crude mortality rate increased with age, for both males and females. Among individuals aged 45-64 years, men experienced 49 deaths with a crude rate of 1.39 per 100,000, while women recorded 276 deaths and a slightly lower crude rate of 1.21 per 100,000. In the 65-74 year age group, deaths rose to 690 among males and 1,640 among females, with corresponding crude mortality rates of 1.81 and 2.07 per 100,000, respectively. The highest burden was observed among those aged 75-84 years, where men experienced 1,348 deaths (crude rate: 3.98 per 100,000) and women recorded 2,455 deaths (crude rate: 4.92 per 100,000). Furthermore, the year-by-year analysis has disclosed minor fluctuations in annual mortality rates for every sex and age group, without a consistent upward or downward trend over time. Additionally, the rates are mostly stable over the six-year period, supporting the decision to pool data for statistical strength and clarity. 

**Figure 1:**
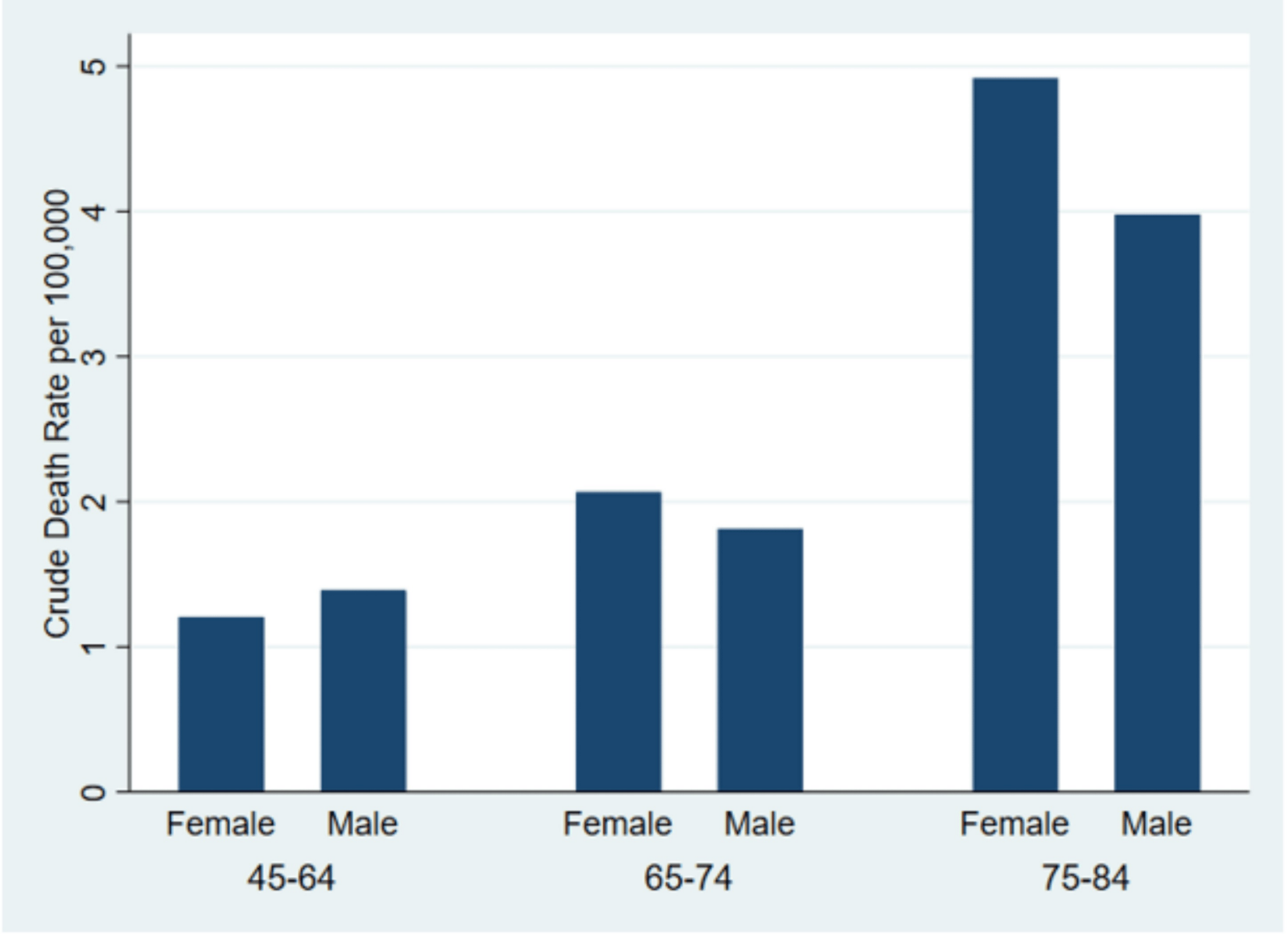
Crude mortality rates for gastroenteritis among adults in the United States by age group and sex, 2018–2023 Crude mortality rates (per 100,000 population) for gastroenteritis of infectious origin (ICD-10 code A09) are shown for adults aged 45–84 years, stratified by sex and age group. Data represent cumulative deaths and population counts from 2018 to 2023, obtained from CDC WONDER. Mortality rates increased with age in both sexes, with women consistently exhibiting higher rates than men in the 65–74 years and 75–84 years age groups.

These findings suggest a clear age-dependent increase in gastroenteritis mortality among adults, with higher crude rates in females than males in the older age groups, as shown in Figure 1 below.

Table 2 below presents the annual number of deaths, corresponding population counts, and crude mortality rates for gastroenteritis (ICD-10 code A09) among U.S. adults aged 45-84 years, stratified by age group and sex from 2018 through 2023.

**Table 2:** Annual gastroenteritis mortality by age group and sex among U.S. adults, 2018–2023 Crude mortality rates (per 100,000 population) for gastroenteritis (ICD-10 code A09) among adults aged 45–84 years in the United States, stratified by sex, age group, and year (2018–2023). N/A- indicate data not available due to suppression or unreliability in the CDC WONDER database. -:Intentionally left blank

Year	Male				Female		
-	Age group	Deaths	Population	Crude rate	Deaths	Population	Crude rate
2018	45-64 years	N/A	N/A	N/A	N/A	N/A	N/A
2018	65-74 years	69	3,873,244	1.78	187	9,915,125	1.89
2018	75-84 years	252	6,220,226	4.05	360	7,937,774	4.54
2019	45-64 years	N/A	N/A	N/A	47	3,790,821	1.24
2019	65-74 years	70	4,182,875	1.67	174	8,672,296	2.01
2019	75-84 years	161	3,900,242	4.13	300	7,089,353	4.23
2020	45-64 years	N/A	N/A	N/A	N/A	N/A	N/A
2020	65-74 years	138	8,275,932	1.67	288	15,755,185	1.83
2020	75-84 years	180	4,222,962	4.26	383	8,433,430	4.54
2021	45-64 years	N/A	N/A	N/A	N/A	N/A	N/A
2021	65-74 years	109	5,351,719	2.04	332	14,285,331	2.32
2021	75-84 years	217	4,924,098	4.41	428	8,237,523	5.20
2022	45-64 years	28	1,767,653	1.58	63	5,676,070	1.11
2022	65-74 years	151	8,326,577	1.81	315	14,271,005	2.21
2022	75-84 years	264	7,113,065	3.71	490	8,931,737	5.49
2023	45-64 years	21	1,758,224	1.19	95	7,644,694	1.24
2023	65-74 years	153	8,064,337	1.90	344	16,497,400	2.09
2023	75-84 years	274	7,493,277	3.66	494	9,303,558	5.31

From the findings in the table above, it’s evident that across all age groups, mortality rates were consistently higher among older adults, particularly those aged 75-84 years. A gradual increase in crude mortality rates was observed over time among females, especially in the oldest age group, with the highest rate of 5.49 per 100,000 recorded in 2022, followed by a slight decrease to 5.31 per 100,000 recorded in 2023. Among males, crude mortality rates in the 75-84 age group remained relatively stable, fluctuating between 3.66 and 4.41 per 100,000. Data for the 45-64 years age group were limited or unavailable for certain years due to data suppression, likely related to small or unreliable counts.

## Discussion

This study investigated the burden and temporal trends of gastroenteritis-related mortality among adults in the United States aged 45 to 84 years between 2018 and 2023, using national mortality data from CDC WONDER. The findings reveal a clear age-dependent increase in mortality, with adults aged 75-84 years experiencing the highest crude death rates. Women consistently exhibited higher mortality rates than men in the 65-74 and 75-84 age groups. While overall mortality rates remained relatively stable for men, an upward trend was observed among women, particularly in the oldest age group. The observed mortality patterns may be attributed to various factors, including divergence in health-seeking behaviors, exposure to long-term care facilities (LTCF), and frailty. Thus, older persons, especially those aged between 75 and 84 years, are increasingly prone to have delayed healthcare-seeking for chronic conditions, mostly as a result of underrecognition of the symptoms, reduced access to urgent care, and mobility limitations, which significantly contribute to the higher fatality rates. Moreover, increased frailty in the age group might heighten susceptibility to complications from gastroenteritis, resulting in adverse outcomes even with treatments. Also, LTCF residents, who form a significant percentage of persons within the oldest cohorts, are at a higher risk for outbreaks and might experience barriers to timely care escalation.

The increasing burden with advancing age is consistent with prior literature, which highlights the greater vulnerability of older adults to infectious gastroenteritis due to age-related immunosenescence, comorbid conditions, and delayed healthcare-seeking behavior [22]. Delays in stool testing and diagnosis can significantly obstruct timely management and treatment in patients with gastroenteritis, especially among older adults who face greater risks of complications and mortality. The delays in stool testing might be a result of several factors, including limited access to diagnostic facilities within certain contexts, clinical prioritizing of immediately life-threatening conditions, logistical challenges in specimen transportation and processing, which may delay and defer gastroenteritis testing. Further, older persons might delay seeking care as a result of underestimation of the severity, mobility difficulties, and dependence on caregivers for transportation, which further compounds the diagnostic delays. 

Quickly identifying the underlying pathogens is crucial for directing appropriate antimicrobial or supportive therapy, especially in certain environments where infections such as *C. difficile *are prevalent. Even with improved diagnostic tools enhancing the accuracy of death reporting, underreporting and mislabeling on death certificates remain persistent challenges, as discussed in this paper. When stool testing is delayed or omitted, healthcare providers may miss the window for effective intervention, resulting in untreated or inadequately managed infections. Delays in diagnosis can lead to increased morbidity and mortality, particularly among aging adults with existing comorbidities or barriers to care [23]. Notably, women in the 75-84 years age group recorded a rise in crude mortality rate from 4.54 per 100,000 in 2018 to 5.31 per 100,000 in 2023. The underlying drivers for this observed sex disparity are not fully understood but may involve differences in health service utilization, chronic disease burden, or care access in late adulthood. Some studies have also suggested that elderly women may be more likely to experience complications from gastrointestinal infections [24, 25].

Temporal analysis across the six-year period showed that gastroenteritis mortality remained a persistent concern among older adults, with modest fluctuations over time. The sharpest increases in crude death rates occurred during 2021-2023, a period coinciding with the COVID-19 pandemic and its aftermath. Although this study only looked at ICD-10 code A09, it's likely that the pandemic's indirect effects, like less access to healthcare, fewer staff in long-term care facilities, and delays in diagnosis or treatment, made gastrointestinal infections worse for vulnerable groups. Given the significant and age-related burden of gastroenteritis mortality in U.S. adults, particularly those aged 75-84, there is a clear public health need to educate the population, especially older adults and their caregivers, on recognizing signs and symptoms of severe dehydration and understanding when to seek hospital care. The study highlights that many gastroenteritis cases, while initially looked at as mild, can rapidly lead to complications like severe dehydration, mainly in elderly individuals with comorbidities or immunocompromised. Furthermore, the rise in mortality rates during and after the COVID-19 pandemic suggests that delays in care-seeking or reduced access to healthcare may exacerbate outcomes. Therefore, teaching people about warning signs like ongoing vomiting, little urine output, dizziness, or confusion could be very important for encouraging early medical help, lowering preventable deaths, and easing the healthcare strain related to infectious gastroenteritis. 

Another notable finding was the limited availability of data for the 45-64 years age group across several years. This was largely due to data suppression in CDC WONDER when mortality counts fall below reporting thresholds or are deemed unreliable. Nonetheless, where data were available (2022-2023), the crude mortality rates in this group were substantially lower than in older adults, reinforcing the age-driven nature of gastroenteritis mortality. These results demonstrate the importance of targeted prevention efforts, especially for adults aged 65 years and older. Strategies may include promoting vaccination against common gastroenteritis pathogens (e.g., norovirus, rotavirus in older adults where applicable), improving hygiene practices in communal settings, enhancing access to early diagnosis and supportive care, strengthening infection control protocols in long-term care facilities, and effectively managing immunity-suppressing comorbidities.

Limitations

This study has several limitations that should be acknowledged. First, the analysis relied on mortality data extracted from CDC WONDER using ICD-10 code A09, which only captures deaths where gastroenteritis was listed as the underlying cause. As a result, cases where gastroenteritis contributed to death but was not recorded as the primary cause may have been missed, leading to potential underestimation of the true burden. Second, the use of crude mortality rates rather than age-standardized rates limits comparability across populations with different age structures, particularly between sexes or across time. Third, data suppression for the 45-64 age group in some years (due to small or unreliable death counts) restricted complete trend analysis for this cohort. Additionally, the “unreliable” and “not applicable” values in CDC WONDER outputs posed analytical challenges and necessitated case-wise data cleaning, which may have introduced selection bias. Thirdly, the database does not provide detailed clinical or sociodemographic variables, such as comorbidities, socioeconomic status, or care setting (e.g., community vs. institutionalized), which limits further interpretation of risk factors and outcomes. Lastly, the ICD-10 code A09 excludes A04.7 (enterocolitis resulting from *C. difficile*), and, as a result, all cases attributable to *C. difficile* have not been captured in the study analysis. The burden of gastroenteritis might, therefore, be underestimated in populations in which *C. difficile* infection is more prevalent.

## Conclusions

This study highlights a significant and age-related burden of gastroenteritis-related mortality among U.S. adults from 2018 to 2023, with the highest crude death rates observed in individuals aged 75-84 years. Women consistently exhibited higher mortality rates than men in the 65-74 and 75-84 age groups, and a notable upward trend in mortality was observed among elderly women over the study period. These findings underscore the ongoing vulnerability of older adults to gastrointestinal infections and the critical need for enhanced prevention strategies, particularly in long-term care settings and among aging female populations. Although platforms, including CDC-WONDER, offer important national mortality data that enable monitoring of trends and estimation of burden, they might fail to effectively capture the granular clinical details, including care settings, comorbidities, and pathogen-specific data, required to guide targeted interventions. Moreover, given the persistent mortality burden and the potential exacerbating effects of healthcare access challenges, especially during the COVID-19 era, targeted interventions are warranted. These may include improving hygiene practices, strengthening infection surveillance, promoting vaccination where applicable, and ensuring timely access to supportive care. Future research should incorporate age-standardized rates, investigate underlying risk factors, and examine regional or racial disparities to provide a more comprehensive understanding of gastroenteritis mortality in the United States.
